# Correlation between Grit score, academic achievement and learning approaches of dental students

**DOI:** 10.1038/s41405-026-00441-0

**Published:** 2026-06-05

**Authors:** Shannu K. Bhatia, Qabirul Abdullah

**Affiliations:** 1 Clinical Reader Hon Consultant, Paediatric Dentistry, School of Dentistry, Cardiff University, Cardiff, UK; 2 Postgraduate Medicine, School of Medicine, Dundee University, Dundee, UK

**Keywords:** Dentistry, Dental education

## Abstract

**Background:**

Importance of non-cognitive skills such as Grit is being increasingly recognised. Grit is the perseverance and passion needed to achieve long-term goals in the face of challenges. This study examines the relationship between Grit scores, academic achievement and learning approaches of dental students

**Methods:**

In this mixed method study, a validated Short Grit Scale was used to determine the Grit score of third year dental students (*n* = 62) and co-related to their assessment score. Relevant statistical analyses were done. Two focus groups were conducted of students with a higher Grit score (HG) and lower Grit score (LG). Thematic analysis was carried out to explore the differences in learning approaches.

**Results:**

Although there was no significant correlation between Grit and academic success, several differences between the higher and lower Grit groups were revealed in the qualitative phase. Major themes were motivation, personality characteristics that equip students to deal with challenges, self-directed learning, and role of peers.

**Conclusions:**

Dental students showed high Grit scores, but these were not associated with academic performance. Higher‑Grit students used deeper, more motivated learning strategies and coped better with pressures, whereas lower‑Grit students were more assessment‑driven. Grit appears to be a desirable construct to develop in students.

## Introduction

Intellectual ability is generally considered to predict academic achievement [1]. Competition and stringent entry criteria, leads to medical and dental school selection of individuals who have previously achieved academic success [2]. It can therefore be assumed that medical and dental students possess a high level of intelligence; however, some students are more successful in their course than others, implying that it takes more than a high IQ to achieve success [3].

Dental students have an intense five-year training period involving challenging academic and clinical learning. University students generally have higher level of stress, feel overwhelmed and ill equipped to handle challenges and are afraid of failing to perform under pressure [4, 5]. Dental students experience anxiety when they perform their first paediatric restorative procedure [6]. Transitioning from preclinical to clinical stage, can cause stress and burnout, related to the academic workload and achievement [7]. Inability to cope with the rigorous training can lead to failure in fully engage or dropping out of the course. The drop-out rate in UK dental schools ranges from 8.4% to 16.8% [8], with consequences not just for the students, but also for the university, the profession and the society.

Both cognitive factors such as intellectual abilities, as well as non-cognitive factors including attitudes, values and personality characteristics play a role towards professional and academic performances [9]. Education providers are increasingly recognising the value of non-cognitive skills like Grit and resilience, as markers of future success in both school and career [10].

Duckworth et al. (2007) [11] explored why some individuals achieve more than others with similar intelligence and suggested that one personal quality, the most prominent leaders in every field had in common was Grit. They defined Grit as “perseverance and passion for long-term goals”, adding that “Grit means working strenuously toward challenges, maintaining one’s effort and not losing interest over time despite setbacks and adversity”. Grit is reported to be associated with professional and academic success independent of IQ [12]. It captures the “ideas of resilience, conscientiousness, self-control and perseverance in one measure”, concepts essential to academic success [13]. People with higher Grit are more successful in accomplishing their goals and excel in competitive environments because they work harder to attain their goals, persevere in the face of hinderances, staying focused on their long-term goals [14].

However, the evidence linking Grit to academic achievement is not entirely consistent and some studies have failed to find a strong or consistent association between them [15], Within the broader personality framework, conscientiousness—one of the traits in the Five-Factor Model of personality [16] has been shown to predict academic achievement as well as, or in some cases better than, Grit [17]. Similarly, a meta-analysis [15] reported substantial overlap between Grit and conscientiousness and concluded that Grit provides little incremental predictive validity for academic outcomes beyond conscientiousness. Nevertheless, perseverance of effort, which is a dimension of Grit, was more likely to forecast outcomes as it is significantly and positively corelated to engagement in educational activities and perceived gain [15].

Grit develops over time, can be influenced by internal and external factors [18] and can ‘wax or wane’ as a reaction to experiences and surroundings [11]. Grit, with its emphasis on long-term stamina is crucial in profession like dentistry and appears to be and desirable construct to develop dental students. Limited studies report on the relation between Grit and dental students’ academic achievement [18, 19] but none explore the relation to learning approaches. This knowledge could inform our practice, impact curriculum development and selection processes, and training and retention of our future workforce.

### Aim

This study explores the correlation between Grit of undergraduate dental students and their academic success and learning approaches.

## Methodology

In this mixed method study, a purposeful sample of all 70, Year 3 dental students at the Cardiff University Dental School, were invited by email to voluntarily participate. A participant information sheet and consent form was provided. Inclusion criteria were enrolment in Year 3 of the BDS programme. Year 3 students were chosen because the students transition to clinics in this busy and intense academic year with increased clinical component and regular assessments. Exclusion criteria included those unwilling to consent to participate.

A validated 8-item Grit–S scale which is shorter and psychometrically stronger than the original 12-item scale was used to measure trait-level perseverance and passion for long-term goals. It is reported to have improved psychometric properties including internal consistency, test–retest stability, consensual validity with informant-report versions, and predictive validity [20]. Ethical approval was obtained and the quantitative data was anonymised. Approval was granted by the University Research Ethics Committee, Cardiff University (Ref 1903a).

### Phase 1: Quantitative phase

The participants were invited to fill out a Grit-S questionnaire in term 1 of third year, and the Grit score was calculated using a 5-point Likert scale with “1” representing “very much not like me” and “5” representing “very much like me.” A corresponding value is assigned between 1 and 5 for each of the eight items. The maximum score is 5 (extremely high Grit) and the lowest score is 1 (not at all Gritty). The students were aware of their Grit scores.

Summative Year 3 Case Reports were used as the indicator of academic achievement as they assess the students’ critical thinking and application of their knowledge in diverse clinical situations. This assessment requires students to write and present a treated case, including diagnosis, treatment planning, evidence‑based discussion, presentation quality, and reflection, and contributes 20% to the overall Year 3 grade. Although multiple assessments should have ideally be used, practical constraints prevented inclusion of the final summative examination. The Case Report was therefore used as the best available measure of academic performance for this study.

The calculated Grit score was used to study its corelation with the academic score of each participant. The Spearman rank co-relation coefficient test was used and simple descriptive statistics and graphs were used to explore the quantitative data initially. Data for Grit and marks were found to be normally distributed.

Grit scores were tested against the central or mid-range value of 3 by using the one-sample *t*-test to determine if they were on the “grittier side” of the scale or not. The relationship between marks and Grit score was found to be linear and residuals were normally distributed about the line and so Pearson’s correlation coefficient was used to quantify and examine this overall relationship. Furthermore, simple linear regression was carried out for all subjects, including males and females separately. Finally, this study contained a single (essentially) continuous covariate, namely, Grit and a single factor, namely, sex and marks were posited to respond to both of these variables. All calculations were carried out by using SPSS V25.

### Phase 2: Qualitative phase

Two separate focus groups discussions were conducted with 12 participants to explore characteristics of higher and lower Grit students because they generate rich data through participant interaction, allowing them to build on each other’s experiences and reveal views that may not emerge individually or through quantitative data [21]. Six participants with the highest scores (HG) and six with the lowest scores (LG) who agreed to participate were allocated to separate focus groups respectively. This allowed each group to be large enough to allow for varying opinions and perspectives but also small enough to allow full individual participation. The six HG students also had highest assessment marks. Six students with Grit score on the lower end of the spectrum who participated in the second LG focus group also had lower assessment marks

The primary investigator facilitated both the focus groups and gave similar prompts to initiate discussions that were audio recorded.  The focus groups ran for approximately one hour each to allow full exploration of the topic till saturation was reached.

For qualitative data analysis, the researcher transcribed the recorded interviews verbatim. A mainly inductive thematic analysis was carried out by systematically identifying and organising insights into patterns codes and themes, using a semantic approach with explicit meanings of the data [22]. The data was repeatedly read actively, analytically, and critically that led to generation of initial codes and then the themes.

## Results

### Qualitative analysis

Sixty-two participants consented, a response rate of 88.6%. The mean Grit score of the participants was 3.54 (range of 2.0 to 4.5). A midpoint of 3 was adjusted for this study. All the students with Grit of 3 and above were placed in higher Grit group (HG) and those below 3 were placed in lower Grit group (LG). Overall, the mean value was 3.54, so the subjects were significantly on the “grittier side” (*P *< 0.001) as shown with one-sample *t*-test.

Academic achievement was measured using marks obtained in case report assessment which ranged from 46% to 78% with a mean of 65.1%.

**Fig. 1:**
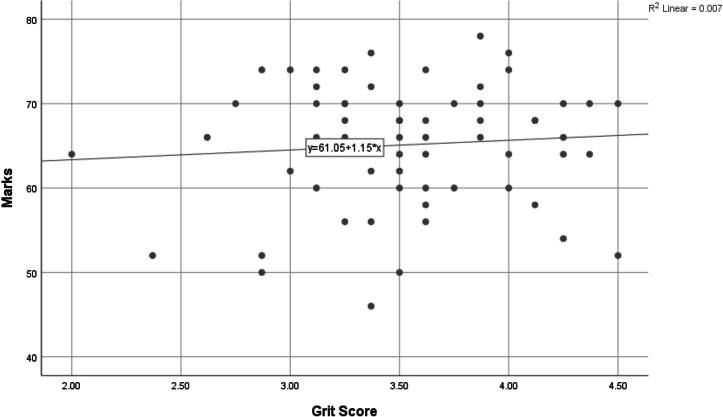
Scatter plot of marks plotted as a function of grit. Shows a very weak liner relationship between two variable grit score and marks obtained.

Pearson’s correlation coefficient, *r*, was used to explore the relationship between the two variables, Grit and academic scores. The scatter plot (Fig. 1) shows a positive yet very weak linear association between the two variables that is not statistically significant (*P* = 0.530). The participants with higher Grit were more likely to have received slightly higher marks than those with lower Grit, however, the size of this effect is very small and not statistically significantly. Indeed, simple linear regression shows that marks increase by only 1.15 mark for each unit increase in Grit score, as shown also by the regression line in Fig. 1. A post-hoc power analysis for linear regression with 2 predictors and sample size of 62 for a medium effect size (Cohenʼs f = 0.15) shows that the power is 76.3%, demonstrating sufficient power to detect a medium effect. The study was, however, underpowered to detect a small effect size should one have been observed hence if an effect exists in the population, it is likely to be small

### Qualitative analysis

**Fig. 2:**
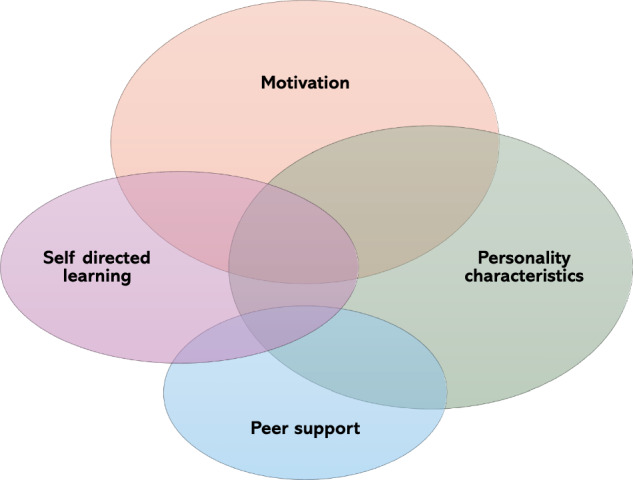
Themes. Themes generated with qualitative analysis include motivation, self directed learning, Personality charcateristics and Peer support.

For thematic analysis, the data was repeatedly read actively and analytically leading to generation of initial codes and then the themes. The quotes start with HG or LG to designate the focus group. Table 1 shows summary of generated codes and themes. These themes were not rigid, as there was a degree of overlap (Fig. 2). For analytical purposes we discuss the themes individually.

**Table 1:** Themes, codes, and example of quotes from lower grit (LG) and higher grit group participants.

Theme	Code	Example quote
**I. Motivation**	Motivation type (Intrinsic / Extrinsic)	LG*: “For me it is just routine of getting up and doing it. I am not really thinking about it, I am going to lectures.”* HG: *to “…. become an all-round better dentist”*
	Avoiding distractions	HG: *“I’ve got this app on my phone called… (to avoid distractions)”*
	Value of low – weight assessments	LG*: “I find it a waste of time when lots of details is given (in seminars) when it is not going to be in exams”*. HG: “*It makes you work hard and keep you on your toes and I like that type of stimulation”*.
**II. Personality characteristics** that equip students to overcome challenges	**Challenges:** Assessment & feedback	LG: “*I don’t really remember sitting it (competency) because I was so stressed”*
	Stress of clinical transition	LG: *“Feel like we did get pushed in the deep end a bit…”* HG: “*You learn a lot as you are doing it”*.
	**Personality Characteristics:** Resilience	HG: *“…I make sure that next time I do better”*.
	Growth mindset	HG: “*I like to problem solve and try to work smart rather than just trying and expecting different results*”.
**III**. **Self-directed learning & Peer assisted learning**	Organisation and planning	LG: *“Bit overwhelmed with how much clinic stuff we had… “* HG: *“…. lists are useful …. helps organise …And also using whiteboards as well, I quite like white boards*”.
	Procrastination	LG: “*If you have had a clinical day the last thing I want to do when I go home is to look at a book. I just want to lie down watch TV”* HG*: “if someone has a filling to do for the first time and you have done a filling, they can give them advice from what you found.”*
	Role of peers	LG*: “helps to know, and which bits don’t come up very often” in assessment.”*

#### Motivation

Although both groups appeared to have different motivations to engage in their learning, the HG participants had more long-term goals, were motivated by meaningful gains like a rewarding career and demonstrated aspects of intrinsic motivation, While LG subjects appeared more passive.

HG1: *“… I want …a job that I love that’s quite challenging and hard, but you can put everything into it and get really rewarding results”*.

LG 3: *“…it is just routine of getting up and doing it. I am not really thinking about it, I am going to lectures.”*

Assessment was a strong motivating factor in both groups. However, LG participants placed more emphasis on the summative and high weight assessments, confessed to last minute preparations and did not appear to value the importance of assessment for learning.

LG 2: “*Cram before exam is the best way forward”*.

LG 3: *“I find it a waste of time when lots of details is given when it is not going to be in exams”*.

Comparatively, HG participants used formative assessments productively to keep themselves “stimulated”, assess their own work, and identify gaps in their knowledge, and focused both on clinical work as well as on long term academic gains. HG looked past immediate challenges and recognised value of effort, revealing an intrinsic motivation

*HG1: “…formative assessment will often force me to revise so it really helps me actually learn the stuff*”.

*HG2: “Lets you know which bits you’re weak on”*.

In addition, HG proactively adopted strategies to avoid distractions when studying. For e.g., some HG students utilised an application that generated points when phones were not used. This generated a healthy competition as they attempted to outdo each other in avoiding unnecessary phone usage. In contrast LG participants had no such strategies.

#### Personality characteristics

Both groups responded emotively to setbacks; however, HG participants analysed their failures and worked harder to overcome the setbacks, showing a growth mindset and resilience. For e.g., two HG students initially faced failure in getting admission to dentistry and undertook it as a second degree. They demonstrated resilience, perseverance, and passion (concepts of Grit), by reapplying for dentistry. *HG 2: “I like to problem solve and try to work smart rather than just trying and expecting different results”*.

HG 4: *“….I’ve had to work a lot harder …things haven’t come as easily …I failed things for the first time, I’ve had to like do resits, but I think that that’s been really good because it’s made me change and I’ve had to work a lot harder”*.

Stress during assessment and clinical transition was more significantly displayed by LG students who felt “nervous” [LG] and started “to panic” [LG].

LG 3: “*I don’t really remember sitting (the exam) … because I was so stressed”*.

The main stress in the HG group was related to workload and feeling “pressure” when peers revised at a faster pace, however, this prompted them to work even harder. Some LG students found it difficult to recall and apply knowledge to clinical scenarios, while other found treating patients (LG1), “*a bit scary*” and felt (LG) “*overwhelmed*”. They were unprepared on clinic and felt they were (LG) “*pushed in the deep end*”.

*LG 1: “I am not really thinking about the theory behind the stuff, I am more just going through the steps”*.

Although both groups adopted Coping strategies inlcuding seeking tutor support and preparing for clinics in advance, the HG students in addition managed their wellbeing by undertaking physical exercises and attempting to strike a work life balance,

*HG 3: “...I …take a step back from work….and don’t try and consume myself – …if I don’t understand something at the time…focus on something else …then come back to it later. I find that if I’ve got a clearer mind then I can understand it a little bit better…I do sports …– I feel like that gives me more of a balance.”*

#### Self- directed learning (SDL) and the Role of Peers

HG students reflected, self-regulated their learning and seemed to have a deeper learning approach. Comparatively, LG students, appearing less proactive and organised, used time ineffectively and did last minute exam preparations due to lack of motivation, workload volume, unavailability of peers and time constraints hence leading to procrastination.

LG 2: “*There is so much that you don’t really know where to start”*

*HG1: “I… look at what… I’m likely to be doing in the session … read up on the theory behind that, write it in a notebook…”*.

LG 1*: “…the last thing I want to do when I go home is to look at a book. I just want to lie down and watch TV”*.

*HG6: “I realised that it's actually best to spend the most time on the things that you don’t understand, and I’ve found that I understand something a lot better if I spend more time reading around it, trying to understand it”*.

Although some LG participants made notes, most of them did no additional reading, and were unaware of, or under-utilised guidelines and handbooks. Instead, they enlisted help of near- peers for strategic learning. On the other hand, HG students took steps to identify and prepare for upcoming clinical procedures and strategically identified sites for relevant information.

LG 4: “*If I am running out of time, I will prioritise what other people have said to learn”*.

HG3*: “I just read through everything as much as I can. I try to rewrite it down – that often helps. If the lectures aren’t details enough, or I don’t understand something, I just use the textbook and that often covers everything I need…”*

Peers and near peers influenced *when, how* and *how much* the participants studied. Both groups implemented peer assisted learning; however, LG used it more for strategic and targeted exam preparations. HG students found it “*really useful”* to discuss new clinical procedures and cases with peers, using the opportunity for peer review and feedback.

*HG “.... if someone has a filling to do for the first time and you have done a filling, they you can give them advice from what you found.”*

In comparison, LG students admitted that peers influenced *what* they studied and enlisted help of peers for strategic exam preparations.

LG 4: “*… (ask peers) to know, and which bits don’t come up very often* (in exams)”. Table 2 summarises differences between higher and Lower Grit students.

**Table 2:** Summary of differences between higher and lower Grit students.

Higher Grit		Lower Grit
Intrinsic	Motivation	Extrinsic
High	Responsibility for patient care	High
High	Value of formative assessment	Low
Yes	Avoiding distraction	None
Yes	Attempting work life balance	None
Deep/ Strategic	Learning approach	Superficial / strategic
Higher	Resilience	High
Higher	Growth mind set	Lower
Higher	Self-directed learning	Lower
Higher	Self-regulation	Lower
Stimulating healthy competition Peer assisted learning	Predominant role of peers	Helping in strategic assessment led learning Peer assisted learning

## Discussion

This study examined the relationship between dental students’ Grit, academic achievement, and learning approaches. The mean Grit score (3.5) was high and consistent with studies in dental, medical, and pharmacy cohorts reporting mean scores between 3.5–4 [23–25]. Dentistry attracts applicants who must demonstrate sustained effort, perseverance, and commitment throughout a competitive admissions process—qualities aligned with Grit and likely contributing to the high scores observed.

No significant correlation was found between Grit and academic performance. Although some studies have shown weak effects of Grit on performance [15, 26] and others have reported more positive associations [25, 27], the overall evidence remains inconsistent. Montas et al. (2001) suggested that dental students with higher Grit achieved higher GPA and class ranks [19]; however, findings across the literature remain mixed. Strategic learning behaviours among lower‑Grit (LG) students may help explain the comparable grades despite differing motivational profiles.

The qualitative findings offered further deeper insight into these behaviours. Motivation plays a crucial role in learning [28], particularly in demanding programmes such as dentistry [29]. Higher‑Grit (HG) students expressed intrinsic motivation [30] engaging in learning for interest, challenge, and long‑term professional development. Lower Grit (LG) students demonstrated more extrinsic drivers, including fear of failure, perceived exam value, and assessment‑driven incentives. Assessment‑driven learning predominated among LG participants, whereas HG students adopted a developmental perspective and welcomed formative assessment. As formative assessment supports learning progression and deeper engagement [31, 32], this aligns with the deeper learning tendencies observed among HG students.

Differences in learning approaches further contributed to these patterns. Surface learning emphasises memorisation [33], deep learning focuses on understanding [34], and strategic learning involves maximising exam performance. HG participants demonstrated characteristics of deep learning, while LG participants showed more surface tendencies but also employed strategic approaches, with LG prioritising topics more likely to come in exams. This strategic behaviour likely contributed to their comparable academic results despite lower Grit.

Stress was common in participants and reflected known challenges in dental education, including academic pressure, early clinical exposure, and patient responsibility [35, 36] Participants reported stress from workload, assessment frequency, and translating theory into practice, consistent with existing literature [37]. LG students reported greater anxiety when linking knowledge to clinical situations and described performing tasks mechanically. As perseverance and consistency of interest are negatively associated with stress [38], these findings are consistent with LG students’ higher stress levels and fewer coping strategies.

Resilience and mindset further differentiated the groups. Resilience—the ability to recover from setbacks [39]—is enhanced by Grit under challenging circumstances [40] and in stressful environments [41]. HG students demonstrated stronger resilience, actively managing setbacks and maintaining balance. Growth mindset—believing intelligence is malleable [42, 43]—also played a role. HG participants expressed growth‑mindset characteristics such as valuing effort, adapting strategies, and viewing setbacks as opportunities [44, 45]. LG students showed fewer such characteristics. The element of persistence and perseverance is thus common to both Grit and a growth mind set which explains why participants with higher Grit showed a growth mindset. A positive relationship between Grit and mindset has been reported in other studies [46]. Students who accept that hard work will improve their abilities are more inclined to exert the effort to develop them, thus Grit is a natural practice for individuals with growth mindset [47]. As Grit and resilience both require overcoming obstacles [11], their combined influence may be important for identifying and supporting at‑risk learners.

Peer learning was valued across groups, supporting understanding, confidence, and critical thinking [48–50]. However, peers also acted as sources of pressure or strategic influence and HG students described this pressure as motivating. While LG participants used peers to identify likely exam topics, reinforcing assessment‑driven study.

Self‑directed learning (SDL) behaviours also differed. SDL involves planning, implementing, and monitoring learning [51, 52], supported by cognitive strategies (e.g., organisation) [53], metacognitive strategies (planning, monitoring, adapting), and motivation [54]. While both groups used cognitive strategies, HG students engaged more in metacognitive processes—time management, preparation for clinical procedures, resource‑seeking, and reflective adjustment. These behaviours align with literature linking Grit to self‑regulation as academic demands intensify [55]. LG students reported feeling overwhelmed, less organised, and more reliant on didactic teaching. A strong positive relationship has been found between self-directed learning and Grit [56], and with self-regulated learning; the students’ engagement in the latter serves as a pathway through which perseverance, an aspect of Grit, is associated with better academic outcomes [57].

Overall, while Grit did not directly predict academic performance, it was associated with intrinsic motivation, deeper learning approaches, lower stress, stronger resilience, and more robust metacognitive strategies. These characteristics may support long‑term development and wellbeing within demanding programmes such as dentistry. Developing Grit‑related attributes—alongside resilience and growth mindset—may therefore help identify and support students at risk of academic or personal difficulties. Systematic reviews also highlight the value of integrating perseverance‑focused activities within curricula to strengthen Grit in higher‑education settings [58, 59]. Education providers can implement evidence‑based approaches to foster Grit. Key strategies include promoting a growth mindset, teaching goal‑setting, encouraging deliberate practice, and supporting students to work constructively through setbacks [60].

### Limitations

Using a single case‑report assessment to represent academic achievement and recruiting students from a single academic year with limited number of participants, are key limitations. A post-hoc sample size calculation revealed the study had reasonable (76.3%) power to detect a medium effect size but was underpowered to detect smaller effect sizes. Therefore, the possibility of a type II error exists, so difference in academic achievement may exist between high- and low-Grit students but if so, it is likely to be small.

Future studies should incorporate multiple formative and summative assessments across several cohorts to provide a more reliable indicator of academic performance. Although the case‑report mark may not fully reflect overall achievement, it enabled the identification of higher‑ and lower‑Grit groups; nevertheless, the quantitative findings should be interpreted with caution. Further research examining the impact of interventions designed to foster Grit in dental students would also be valuable.

## Conclusions

Dental students showed high Grit scores, though no significant association with academic performance was found. Qualitative findings indicated that higher‑Grit students adopted deeper learning approaches, were intrinsically motivated, and demonstrated greater resilience and self‑regulation. Lower‑Grit students tended toward assessment‑driven, strategic learning. All students reported considerable academic and clinical pressures, but higher‑Grit students appeared to manage these demands more adaptively. While not linked to academic scores, these patterns suggest Grit may influence how students engage with learning and cope with the challenges of dental training.

## Supplementary information

Appendix

## Data Availability

Anonymised raw data is available.
