# Supplementary material for: Correlation between Grit score, academic achievement and learning approaches of dental students
Source: BDJ Open. 2026 Jun 5;12:59. doi: 10.1038/s41405-026-00441-0 (PMC13241485; doi:10.1038/s41405-026-00441-0)
Supplement: Supplementary file 1 — Appendix [file 41405_2026_441_MOESM1_ESM.docx]

Appendix

The Grit measure survey uses a 5-point Likert scale with “1” representing “very much not like me” and “5” representing “very much like me.”  A corresponding point value is assigned between 1 (low) and 5 (high) for each of the eight items. Question numbers 2,4,7 and 8, detects a high grit in a participant. The remaining (1, 3, 5, and 6) detect low grit. Individual's grit score is derived by averaging point values for each item. Once adjusted, all the points are added up and then divided by 8 to the give the Grit score for the individual. The maximum score is 5 (individual is extremely gritty /has extremely high grit) and the lowest score is 1 (not gritty). The midpoint is 2.5; any score above that is considered a high score. This Appendix shows the Grit scale and values of each item.

 Short Grit Scale

Short Grit Scale

**Student number***…12345*

*Directions for taking the Grit Scale: Please respond to the following 8 items.  Be honest – there are no right or wrong answers!*

1. **New ideas and projects sometimes distract me from previous ones. ***

q Very much like me

q Mostly like me

q Somewhat like me

q Not much like me

q Not like me at all

2**. Setbacks don’t discourage me.**

q Very much like me

q Mostly like me

q Somewhat like me

q Not much like me

q Not like me at all

3**. I have been obsessed with a certain idea or project for a short time but later lost interest. ***

q Very much like me

q Mostly like me

q Somewhat like me

q Not much like me

q Not like me at all

4. **I am a hard worker.**

q Very much like me

q Mostly like me

q Somewhat like me

q Not much like me

q Not like me at all

5**. I often set a goal but later choose to pursue a different one. ***

q Very much like me

q Mostly like me

q Somewhat like me

q Not much like me

q Not like me at all

6**. I have difficulty maintaining my focus on projects that take more than a few months to complete. ***

q Very much like me

q Mostly like me

q Somewhat like me

q Not much like me

q Not like me at all

7**. I finish whatever I begin.**

q Very much like me

q Mostly like me

q Somewhat like me

q Not much like me

q Not like me at all

8**. I am diligent.**

q Very much like me

q Mostly like me

q Somewhat like me

q Not much like me

q Not like me at all

**Scoring:**

1. For questions 2, 4, 7 and 8 assign the following points:

5 = Very much like me

4 = Mostly like me

3 = Somewhat like me

2 = Not much like me

1 = Not like me at all

2. For questions 1, 3, 5 and 6 assign the following points:

1 = Very much like me

2 = Mostly like me

3 = Somewhat like me

4 = Not much like me

5 = Not like me at all

Add up all the points and divide by 8. The maximum score on this scale is 5 (extremely gritty), and the lowest score on this scale is 1 (not at all gritty).
